# Preparation and Evaluation of MXene/Graphene-Integrated Cellulose Aerogel Composite for Self-Heating Thermoregulation in Athletic Warm-Up Optimization

**DOI:** 10.3390/gels12040320

**Published:** 2026-04-08

**Authors:** Xinran Qian, Lanqing Ling, Dengyun Xu, Jialu Lu, Haohan Liu, Meng Yuan, Tianfeng Lu, Lejun Wang, Ai Du, Lili Qin

**Affiliations:** 1 Sports and Health Research Center, Department of Physical Education, Tongji University, Shanghai 200092, China; qianxinran@tongji.edu.cn (X.Q.); linglanqing@tongji.edu.cn (L.L.); 2433581@tongji.edu.cn (D.X.); liuhaohanjojo@163.com (H.L.); 13751166455@163.com (M.Y.); 02110@tongji.edu.cn (T.L.); wlj0523@163.com (L.W.); 2 Shanghai Key Laboratory of Special Artificial Microstructure Materials and Technology, School of Physics Science and Engineering, Tongji University, Shanghai 200092, China; jialu21@tongji.edu.cn

**Keywords:** MXene, aerogel, phase change material, solar energy, warm-up transition phase, physical education

## Abstract

A warm-up is a critical procedure in sports science for enhancing muscular performance and optimizing subsequent athletic activities. However, the physiological and athletic performance effects of a warm-up are often transient, diminishing rapidly during the period of inactivity after the warm-up, which is known as the warm-up transition phase. In this study, a multi-functional thermoregulation wearable composite film of graphene–MXene–bacterial cellulose/polyethylene glycol (G-M-BC/PEG) was developed by integrating MXene (a two-dimensional material with good photothermal conversion performance) and graphene into a bacterial cellulose aerogel framework, subsequently impregnated with polyethylene glycol (PEG-2000). The film showed stable structure, efficient solar photothermal conversion and storage (SPCS), and improved mechanical properties. Under 1 sun irradiation, the optimized G-M-BC/PEG wearable film showed excellent SPCS performance, sustaining a temperature plateau of 38–40 °C for 10 min after the xenon lamp was switched off under 1 sun irradiation, with a leakage rate of only 5.32% after five cycles. By constructing a biomimetic sports human body model, the composite aerogel was shown to significantly elevate muscle surface temperature and effectively mitigate heat loss during the transition phase. In the warm-up effectiveness and sports performance tests, the wearable film improved 200 m sprint performance by 0.8% ± 0.4% (*p* = 0.039). It also maintained subjective thermal sensation during the warm-up transition phase, with no significant decline at 5 or 10 min after the warm-up and a significant decrease only at 15 min (*p* = 0.02), while thermal comfort remained stable, suggesting improved neuromuscular readiness. This research provided a novel strategy for the fabrication of advanced aerogel-based wearable devices aimed at precision thermal management and athletic performance optimization.

## 1. Introduction

Warm-up activities are the core content of the preparatory part of exercising [1] which increase muscle and core temperature and improve nerve conduction within the peripheral and central nervous systems [2,3], which ultimately improves athletic performance [4]. When athletes adopt a rational warm-up activity, the injury prevention benefits they obtain are positively correlated with the degree of warm-up [5,6]. Some studies refer to the time between the end of the warm-up activity and the beginning of the game as the transition phase, i.e., the warm-up transition phase [7,8,9]. In the period of inactivity following a warm-up, the benefits from the warm-up are lost and bring about a decrease in athletic performance [1]. Athletes usually need to experience a warm-up transition phase of 10 to 30 min before the start of a competition, and a prolonged transition period reduces core and muscle temperature [8,9]. At the same time, outdoor physical training in the fall and winter seasons is combined with cold and windy weather and other unavoidable weather factors, which can negatively affect athletic performance and exacerbate the loss of the warm-up effect during the warm-up transition phase [10].

Initially, hot showers were employed to counteract the drop in muscle and core temperatures following preparatory activities prior to formal exercise in programs such as swimming [11], but logistical and practical issues have made this method difficult to use in most exercise scenarios [12]. Instead, recent research has developed methods for employing smart wearables to keep muscle and core temperatures elevated during the transition phase between the warm-up and subsequent exercise performance. Conventional heat-maintenance strategies during the post-warm-up transition period mainly depend on passive insulation and external heat sources, such as heating garments and disposable warming packs, to reduce heat loss and help preserve elevated muscle temperature before exercise performance [13]. One study significantly reduced the drop in muscle temperature in the transition phase while increasing the athlete’s final peak power output through the use of heated sweatpants during the build-up period prior to a sprint cycling race [14]. In addition, many similar passive heated garments and life jackets have been used to regulate core and muscle temperatures during the warm-up transition phase and have improved athletic performance and reduced athletic injuries in sports such as bobsledding [15], rugby [16], swimming [17,18] and rowing [19]. Therefore, the use of wearable devices as passive heating devices during the transition period is effective in maintaining higher muscle temperatures obtained during an active warm-up, avoiding muscle stickiness and enhancing muscle elasticity, thus enhancing the warm-up effect and improving athletic performance during the exercise period [20].

Among the various photothermal materials that have been developed, such as carbon-based materials [21], noble metal nanoparticles [22], conjugated polymers [23] and metal–organic frameworks [24], MXene stands out due to its high aspect ratio, atomically thin layer thickness, excellent photothermal properties, low toxicity, etc. [25,26,27]. MXenes are a class of two-dimensional transition-metal carbides/nitrides/carbonitrides with the general formula M_n+1_X_n_T_x_, and their surfaces are typically terminated by functional groups such as –O, –OH, and –F after selective etching of MAX phases [28,29]. This layered structure and rich surface chemistry provide MXene with good hydrophilicity and interfacial compatibility in composite systems. It has been shown that MXene has a wide range of light-absorbing wavelengths and an internal light-to-heat conversion efficiency of nearly 100%, which allows it to absorb and convert clean, renewable solar energy to generate thermal energy without the need for additional energy [30]. In addition, in the aerogel structure, MXene easily forms a thin film-like shape, which has excellent flexibility, comfort, breathability, photothermal conversion, UV shielding, and antimicrobial properties [31,32]. Its abundant surface chemical groups provide a pathway for the formation of hybrid aerogels and offer a great potential for the construction of MXene-based aerogel structures with high photo- and thermo-conversion properties [33], which can provide a structural basis for the subsequent impregnation of phase change materials. To further enhance the photothermal conversion efficacy of wearable films, graphene was chosen to be synergized with MXene in this study. Recent MXene and graphitic material-based stretchable systems have shown that highly deformable conductive interfaces can support real-time functionality under dynamic mechanical conditions, highlighting the importance of flexibility in skin-contact wearable platforms [34,35]. Graphene has unique optical and electrical properties [36] and exhibits good photothermal conversion performance and photo-responsive properties [37]. Bacterial cellulose (BC) aerogel was selected as a substrate for the preparation of MXene-based thermoregulation wearable devices. The thin film layer formed by MXene combined with BC has excellent photothermal conversion, thermal insulation and heat preservation [30]. Therefore, combining bacterial cellulose with MXene and graphene to prepare aerogels can provide good photothermal conversion performance while obtaining a certain thermal insulation effect and mechanical support, further slowing down the heat release of the phase change material and reducing the leakage of the phase change material. A PCM (phase change material) is a substance that absorbs or releases the heat of a phase change during the phase transformation it undergoes, thereby storing energy and regulating and controlling the temperature of the environment [38]. Polyethylene glycol (PEG) is a more typical medium- to low-temperature phase change material in the phase change field, which can easily be compounded with other polymers [39]. Under light conditions, it can play the role of melting and absorbing heat to protect the safety of human skin during exercise, while it can also continue to release heat through a heat-absorbing and melting action under the condition of losing light, so that the smart thermoregulation wearable device can maintain a stable temperature platform within a certain period of time to meet the demand for human body heat management in the transition phase of warming up [40,41].

Therefore, it is necessary to adopt scientific methods to help athletes through the warm-up transition phase in order to maintain the high core and muscle temperatures obtained during warm-up activities, so as to optimize athletic performance, avoid athletic injuries, and improve the efficiency of sports training. In this study, thermoregulation wearable devices of G-M-BC/PEG composite aerogels were prepared by combining MXene, graphene, BC, and PEG, which can intelligently regulate temperature while efficiently converting light and heat and play a role in maintaining or enhancing the warm-up effect and athletic performance in physical training. In addition, this study also comprehensively evaluates the overall application effect of intelligent temperature-regulating wearable devices in the warm-up transition phase of sports training through material characterization and sports human body experiments to provide a certain theoretical and practical basis for the future application of intelligent wearable devices in the field of sports training.

## 2. Results and Discussion

### 2.1. Analysis of G-M-BC/PEG Composite Aerogels

#### 2.1.1. Microstructures

**Figure 1:**
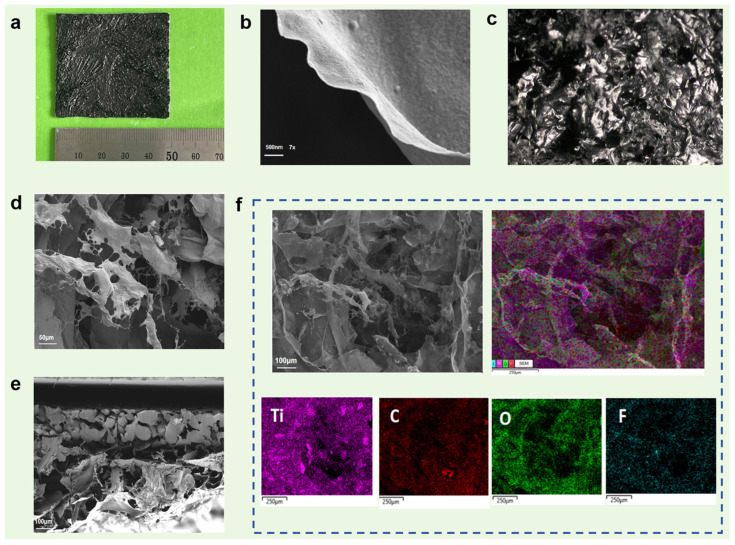
Photograph and microstructures of G-M-BC/PEG composite phase change films. (**a**) Photograph of G–MXene–BC/PEG composite phase change films. (**b**) Magnified 7× optical microscope image of G–MXene–BC aerogel film surface. (**c**) SEM image of MXene powder. (**d**,**e**) SEM images of upper surface and cross section of G–MXene–BC aerogel films. (**f**) EDS images of G–MXene–BC aerogel film.

As shown in Figure 1a, the G–MXene–BC/PEG composite phase films exhibit good macroscopic integrity, suggesting successful film formation and uniform PEG incorporation. Figure 1b further reveals a relatively uniform and smooth surface morphology of the G–MXene–BC aerogel film without obvious cracks or phase separation. The SEM images of the samples (Figure 1c–e) show that MXene is well-compounded with GO and BC, and the compound has a large pore size and good spatial structure after freeze-drying. As can be seen from the cross-sectional view, the G–MXene–BC aerogel exhibits a multilayer structure with the polyethylene film used as a substrate. In addition, the EDS mapping results (Figure 1f) confirm the homogeneous distribution of the key elements, further verifying the successful incorporation of MXene into the GO–BC matrix.

#### 2.1.2. Characterization Properties

**Figure 2:**
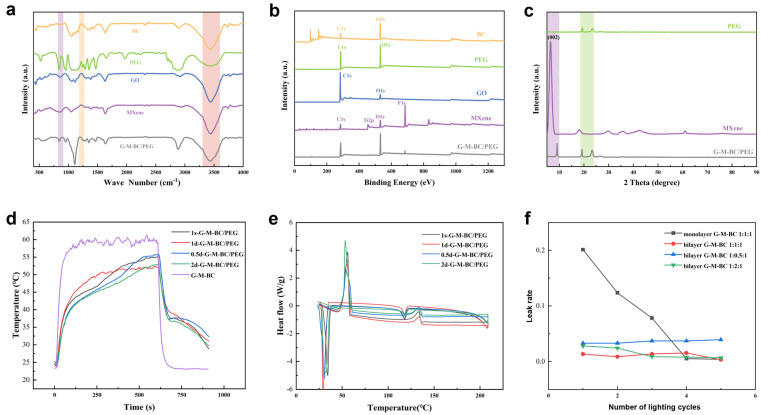
(**a**) FTIR and (**b**) XPS analyses of BC, PEG, GO, MXene and the G-M-BC/PEG aerogel. (**c**) XRD analyses of PEG, MXene, and the G-M-BC/PEG aerogel. (**d**) Structural photothermal conversion and storage curves of G-M-BC/PEG composite phase change films with different mass ratios. (**e**) DSC curves of different mass ratios of G-M-BC/PEG composite phase change films. (**f**) Leakage rates of five cycling experiments of films with different ratios at one solar light intensity.Color coding: (**a**–**c**) Orange: BC, green: PEG, blue: GO, purple: MXene, gray: G-M-BC/PEG; (**d**) Black: 1s-G-M-BC/PEG, red: 1d-G-M-BC/PEG, blue: 0.5d-G-M-BC/PEG, green: 2d-G-M-BC/PEG, purple: G-M-BC; (**f**) Black: monolayer G-M-BC 1:1:1, red: bilayer G-M-BC 1:1:1, blue: bilayer G-M-BC 1:0.5:1, green: bilayer G-M-BC 1:2:1.

In order to analyze the attachment between MXene, GO, PEG and BC, the FTIR spectra are given in Figure 2a. The FTIR spectra of each component reveal a broad absorption peak (3400–3600 cm^−1^), corresponding to the -OH stretching vibration, which indicates abundant -OH functional groups on their surfaces, contributing to the adsorption of components in the BC. For MXene, the characteristic absorption peaks at 866 cm^−1^ and 1231 cm^−1^ are assigned to stretching vibrations of Ti-O and C-O. In addition, no significant new peaks were found in the G-M-BC/PEG spectra, suggesting that no chemical interactions occurred except those based on physical absorption such as van der Waals forces and intermolecular hydrogen bonds between the abundant surface functional groups.

The X-ray photoelectron spectroscopy (XPS) analysis of the components provides insights into their chemical composition. In Figure 2b, the XPS survey scan of MXene confirmed the presence of C, Ti, O, and F; at the binding energies of 285 eV, 455 eV, 523 eV and 666 eV, no new peaks appeared for G-M-BC/PEG. The results show agreement with FTIR analyses, which suggests morphological stability during phase transitions.

The X-ray diffraction (XRD) survey spectra of the MXene, PEG and G-M-BC/PEG film are presented in Figure 2c. MXene exhibited a wide characteristic peak at approximately 6.32°, which is attributed to the crystal structure (002) around the crystal plane, indicating that the MAX material was successfully etched and the layered structure appeared. The XRD pattern of PEG showed two specific sharp diffraction peaks at 19.1° and 23.4° of the 2θ angle. There were no new peaks in the XRD spectrum of the G-M-BC/PEG film. The diffraction peaks around 19.1° and 23.2° are the characteristic peaks of PEG. The typical and characteristic diffraction peak of MXene is presented at 9°.

Simulating sunlight intensity illumination (Figure 2d), there was a significant decrease in the maximum temperature of the composite phase change material containing PEG-2000, and the maximum surface temperature of the samples gradually increased with the increase in MXene concentration. The maximum temperatures of the 1d- and 2d-G-M-BC/PEG films reached 52.4 °C and 52.9 °C, respectively, which indicates that the G-M-BC aerogel composite with PEG can still exert its excellent photothermal conversion properties.

The DSC test results showed that with the increase in the mass fraction of MXene, the melting enthalpy and crystallization enthalpy of the G-M-BC/PEG composite phase change films roughly followed a regular upward trend and showed higher peak temperatures of phase change and a more concentrated range of phase change temperatures (Figure 2e). Therefore, it can be inferred that a layered MXene structure with a larger mass fraction and the bilayer aerogel structure can provide excellent spatial structural conditions for the impregnation of phase change materials, which allows PEG to perform a better heat storage function.

Leakage rate tests revealed that monolayer MXene-based aerogel-encapsulated phase change films leaked more than 20% of the phase change material in the first cycle. Therefore, a bilayer aerogel-encapsulated structure was adapted. As shown in Figure 2f, after five light cycles, the leakage rate of the composite phase change films with bilayers was significantly lower than those with monolayers, while the total PEG leakage rate of 1d-G-M-BC/PEG was the lowest at 5.32%. This indicates that the G-M-BC ratio used for the spatial structure formed by the bilayer aerogel is the best ratio for the impregnation and encapsulation of PEG.

#### 2.1.3. Mechanical Properties

**Figure 3:**
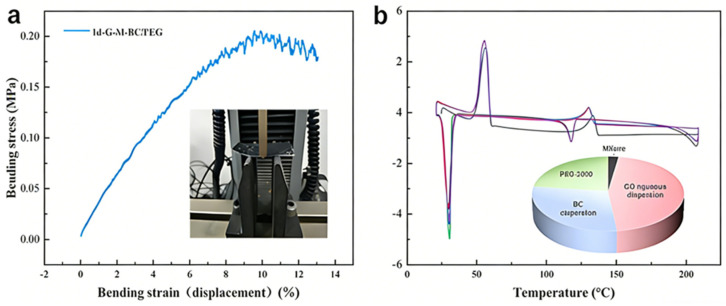
(**a**) Stress–strain curves of 1d-G-M-BC/PEG composite phase change film. (**b**) DSC plots of the 1d-G-M-BC/PEG film after undergoing five heat-absorbing and exothermic cycles.

In addition, the 1d-G-M-BC/PEG film exhibits a notable elastic modulus of 3.21 MPa, accompanied by a bending stress at the peak load of 0.208 MPa (Figure 3). This attribute allows for adaptation to large deformations of the skin and muscles on the surface of the body and to various external stimuli, which provides an excellent mechanical foundation for its use as a wearable device. As a result, the G-M-BC/PEG composite phase change film shows not only low leakage rates and high thermal storage properties but also excellent cycling stability and flexibility properties.

In summary, the preparation process of HCL-LiF-etched MXene in this study is easy to realize, and the bilayer G-M-BC/PEG composite aerogel for the warm-up transition phase in sports exercise can be safely prepared at room temperature using simple equipment. The G-M-BC/PEG composite aerogel is prepared with high efficiency and has a wide range of applicability. The 1d-G-M-BC/PEG thermoregulating wearable film has good photothermal conversion performance and heat storage performance and at the same time demonstrates better mechanical properties and reusability in the subsequent performance tests. It meets the needs of wearable films to fit human muscles, maintain body temperature, reduce heat dissipation and be reusable and provides a good material basis for the application of wearable films in sports teaching. This study followed up with an experimental study of the human body based on the warm-up transition phase of a 200 m sprint to explore more specific practical strategies for the application of G-M-BC/PEG composite aerogel in sports exercise.

### 2.2. Solar Thermal Effect of Aerogels

**Figure 4:**
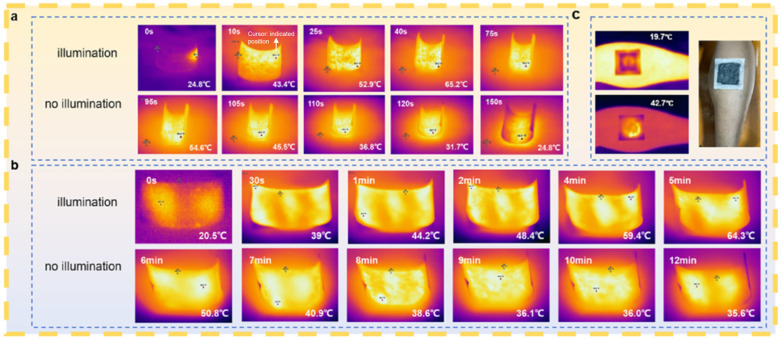
Infrared thermal images of the warming time of illuminated lamps versus the cooling time after turning off the lamps under one solar illumination condition: (**a**) G–MXene–BC aerogel film; (**b**) bilayer G–MXene–BC/PEG composite phase change films; (**c**) these films applied on calf muscle belly. Cursor: indicated position.

During the temperature rise caused by xenon lamp irradiation, there is a “plateau” in the light temperature curve, which indicates that the photothermal effect produced by MXene and GO successfully increased the temperature, demonstrating the role of PEG in heat absorption and melting (Figure 4). The temperature of the composite phase change material decreased sharply as the experiment continued for 10 min after stopping the xenon lamp irradiation, but the trend of the temperature decrease became flat around 38–40 °C. This result indicates that the introduction of the phase change material into the MXene-based aerogel film can effectively slow down heat dissipation after losing the light condition. These results are consistent with recent reports on MXene-based phase change composites, which have demonstrated effective photo-to-thermal conversion and enhanced thermal management through the incorporation of MXene into porous supporting matrices, such as biomass-derived scaffolds or aerogel frameworks [27,38].

### 2.3. Analysis of Warm-Up Effectiveness and Sports Performance Indicators

**Figure 5:**
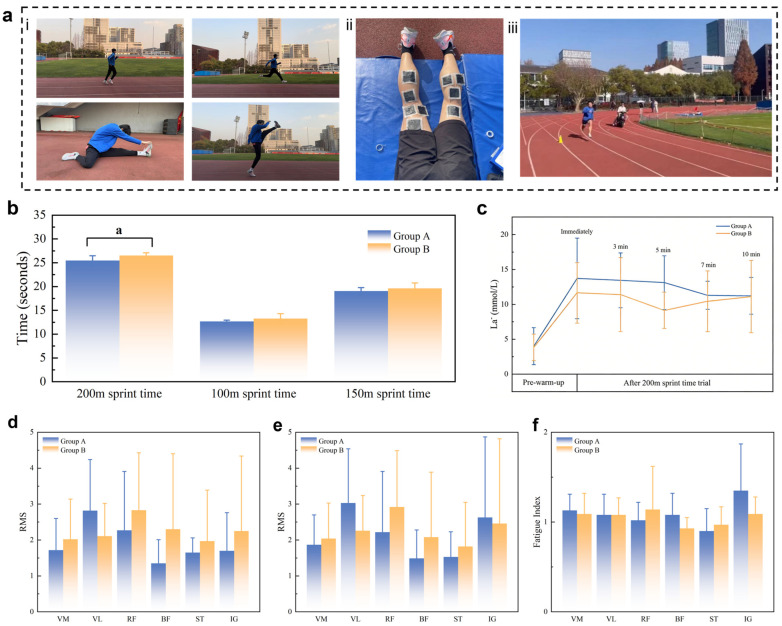
(**a**) Exercise model. (**i**) 30 min standardized warm-up. (**ii**) 15 min transition phase. (**iii**) 200 m sprint time trial. (**b**) 200 m sprint time trial performance times in both conditions, a: significant difference between groups (*p* ≤ 0.05). (**c**) Change in blood lactate (La¯) from baseline (pre-warm-up) to 10 min after a 200 m sprint, for both conditions. (**d**) RMS during the EMG active phase. (**e**) RMS during the EMG fatigue phase. (**f**) Statistical histograms of FI for each muscle. (**b**,**c**) Data are presented as mean ± standard deviation. Results are significantly different between groups when *p* ≤ 0.05. Detailed original data are provided in Tables S2–S6.

This study constructed an exercise model of a 30 min standardized warm-up followed by a 15 min transition phase and a 200 m sprint time trial (Figure 5a). The skin temperature (Tskin) of the muscle surface was recorded at six time points by infrared thermography (FOTRIC 225S), with the measuring point set at the center of the popliteal fossa of the right leg. As shown in Table S1, via the warm-up transition intervention in Group A, the Tskin was significantly and sustainedly increased from pre warm-up during the whole transition phase (after warm-up 5 min, *p* = 0.04; 10 min, *p* = 0.008; 15 min, *p* = 0.002). Therefore, the Tskin at 10 min (*p* = 0.021) and 15 min (*p* = 0.007) after the warm-up was significantly increased by 1.96 °C compared to that measured immediately after the warm-up. The Tskin decreased after the 200 m sprint in both conditions, but it was significantly different between other time points in the warm-up transition in Group B. No significant changes occurred during the transition phase in Group B. There was no significant interaction effect between the two groups (*p* = 0.165 > 0.05).

#### 2.3.1. Thermoregulation

As reported above, subjects in Group A exhibited a significant and sustained elevation in muscle surface temperature throughout the 15 min warm-up transition phase. Specifically, Tskin at 10 min (*p* = 0.021) and 15 min (*p* = 0.007) post warm-up remained 1.96 °C higher than values recorded immediately after the warm-up. In contrast, Group B showed a progressive decline in Tskin during the same period, with no statistically significant differences among post-warm-up time points. No significant group-by-time interaction was observed (*p* = 0.165).

Subjective thermal sensation, assessed using the ASHRAE seven-point scale, increased significantly after the warm-up in both groups and remained elevated throughout the transition in Group A. In Group A, thermal sensation at 5 min (*p* = 0.451) and 10 min (*p* = 0.104) did not differ from the immediate post-warm-up value, but a significant decrease was noted at 15 min (*p* = 0.02). In Group B, no significant changes were detected across the four post-warm-up time points, and the interaction between group and time was not significant (*p* = 0.057). Thermal comfort, measured on a five-point scale, remained stable and comparable between groups throughout the test (interaction *p* = 0.309), with scores ranging between 0 (comfortable) and 1 (slightly uncomfortable).

Together, these results indicate that the G-M-BC/PEG composite aerogel effectively preserved the heat gained during the active warm-up, maintaining higher skin temperature and enhanced thermal sensation without compromising comfort.

#### 2.3.2. Sports Performance Enhancement

Performance times: As shown in Figure 5b, Group A achieved a significantly faster 200 m sprint time than Group B, with an improvement of 0.8% ± 0.4% (*p* = 0.039). No significant differences were observed for the 100 m (*p* = 0.117) or 150 m (*p* = 0.109) split times, suggesting that the performance benefit emerged primarily in the latter part of the sprint.

Heart rate: Post-sprint heart rate was significantly elevated compared to all earlier time points in both groups (*p* < 0.05). In Group A, heart rate remained significantly higher than pre-warm-up values at 10 min (*p* = 0.015) and 15 min (*p* = 0.024) after the warm-up, whereas in Group B, heart rate returned to baseline more rapidly. The group-by-time interaction was not significant (*p* = 0.908).

Blood lactate: Blood lactate concentration increased markedly after the 200 m sprint in both groups, consistent with a heavy reliance on glycolysis. As illustrated in Figure 5c, the initial rate of lactate accumulation was faster in Group A, suggesting enhanced glycolytic flux. However, the overall lactate clearance within 10 min post-sprint was similar between groups, and no significant interaction was found (*p* = 0.262).

Rating of perceived exertion: RPE remained near the baseline throughout the transition phase in both groups and rose sharply only after the sprint (*p* < 0.05), with no significant interaction (*p* = 0.359).

Collectively, these findings demonstrate that wearing the thermoregulation device led to a meaningful improvement in 200 m sprint performance, accompanied by a more rapid glycolytic response, while cardiovascular and perceptual responses remained similar between conditions.

Previous studies using external heating garments have demonstrated that maintaining higher body or muscle temperature during this interval can attenuate thermal decline and support performance-related outcomes. Similarly, in the present study, the G-M-BC/PEG wearable material helped preserve thermal sensation and muscle surface temperature during the warm-up transition period [3].

#### 2.3.3. Neuromuscular Activation

Surface EMG signals were recorded from six lower limb muscles during both the active and fatigue phases of the 200 m sprint. Electrodes were placed on the vastus medialis (VM), vastus lateralis (VL), rectus femoris (RF), biceps femoris longus (BF), semitendinosus (ST), and medial gastrocnemius (IG) following standard skin preparation.

Active phase: As shown in Figure 5d, the ranking of RMS values differed between groups. In Group A, the VL showed the highest activation (2.82 ± 1.42), followed by the RF (2.27 ± 1.64), whereas in Group B, the RF was most active (2.83 ± 1.60), followed by the BF (2.30 ± 2.10). With the exception of the VL, muscle activation tended to be higher in Group B, possibly reflecting a compensatory strategy to maintain force output under cooler muscle conditions. Although no statistically significant intergroup differences were found for any muscle, the predominant role of the VL in Group A suggests that optimal muscle temperature may facilitate more efficient neuromuscular recruitment.

Fatigue phase and fatigue index: During the fatigue phase (Figure 5e), RMS values increased across all muscles in both groups, indicating greater neural drive to sustain power output. In Group A, the VL remained the most activated muscle (3.03 ± 1.50), followed by the IG (2.63 ± 2.24); in Group B, the RF (2.92 ± 1.57) and IG (2.46 ± 2.36) were predominant. The fatigue index (FI) for each muscle is shown in Figure 5f. Notably, the IG in Group A exhibited sustained high activation, contributing to explosive force production despite overall fatigue. This pattern implies that the thermoregulation device may enhance fatigue resistance in key propulsive muscles, potentially lowering the risk of fatigue-related injury.

Taken together, these EMG data indicate that while no significant differences in individual muscle RMS values were detected, the observed trends—particularly the prominent role of the VL in the active phase and the sustained contribution of the IG during fatigue—suggest that maintaining elevated muscle temperature may promote more efficient neuromuscular function and delay fatigue.

#### 2.3.4. Summary of Multidimensional Effects

In this study, the G-M-BC/PEG composite aerogel demonstrated three interrelated functional benefits during the warm-up transition phase:

1. Thermoregulation: The material effectively elevated and sustained muscle surface temperature, preserving the thermal benefits of an active warm-up.
2. Sports performance enhancement: It significantly improved 200 m sprint time and accelerated initial blood lactate accumulation, indicating enhanced explosive power and glycolytic energy turnover.
3. Neuromuscular activation: Although no statistically significant differences in EMG parameters were observed, the observed trends—such as the dominant role of the VL in the active phase and the sustained contribution of the IG during fatigue—suggest that the device may facilitate more efficient muscle recruitment and delay fatigue, with potential implications for injury risk reduction.

These findings support the hypothesis that the 1d-G-M-BC/PEG composite aerogel influences athletic performance primarily through thermal mechanisms (reducing muscle viscosity, improving hemodynamics, and enhancing glycolytic efficiency). Future studies with larger sample sizes or refined device designs are warranted to further elucidate the underlying mechanisms and to confirm the observed neuromuscular trends. It should be noted that the current study represents an initial exploration of the application of this thermoregulation material in a sports context, and the reproducibility of the performance findings warrants further investigation with larger sample sizes and independent cohorts in future studies.

## 3. Conclusions

In summary, this study explored and determined the preparation method and optimal formulation of G-M-BC/PEG composite aerogel by experimental methods. Among them, the 1d-G-M-BC/PEG thermoregulation wearable film demonstrated photothermal conversion performance and heat storage performance. It also exhibited better mechanical properties and reusability in the subsequent performance test, which meets the needs of wearable devices to fit human body muscles, maintain body temperature, reduce heat loss and enable reuse, providing a solid material foundation for further applications of wearable devices in sports exercise. Additionally, in a human experiment conducted during the warm-up transition phase, the 1d-G-M-BC/PEG composite aerogel could maintain the warm-up effect of athlete subjects to a certain extent and significantly enhance the athletic performance of athletes in a 200 m sprint. In conclusion, we believe that the application of the thermoregulation wearable device in the warm-up transition of athletes’ sports training is a useful and effective attempt, and it holds great potential for broader adoption and application in the field of sports exercise. As this study serves as a preliminary investigation into the application of G-M-BC/PEG composite aerogels in sports performance, future work will focus on validating the reproducibility of these findings through larger-scale, multi-center trials and further elucidating the underlying mechanisms (Table S7).

## 4. Materials and Methods

### 4.1. Preparation and Characterization of G-M-BC/PEG Composite Aerogels

#### 4.1.1. Materials

The Ti_3_AlC_2_ nanosheets (purity 98–99%) were purchased from Jilin 11 Technology Co., Ltd., Changchun, China. The graphene oxide aqueous dispersion (GO, 5 mg/mL) was purchased from Nanjing Research Institute of Sinosteel (Nanjing, China). The bacterial cellulose dispersion (BC, 0.8%) was purchased from Guilin Qihong Technology Co., Ltd., Guilin, China. Breathable film was purchased from Dongguan Wells Technology Co., Ltd., Dongguan, China. Lithium fluoride (LiF, 99%) was supplied by Shanghai Aladding Biochemical Technology Co., Ltd., Shanghai, China. Hydrochloric Acid (HCl) and PEG-2000 were supplied by National Pharmaceutical Group Chemical Reagent Co., Ltd., Shanghai, China. Deionized water was used throughout.

#### 4.1.2. Synthesis of MXene Nanosheets

**Figure 6:**
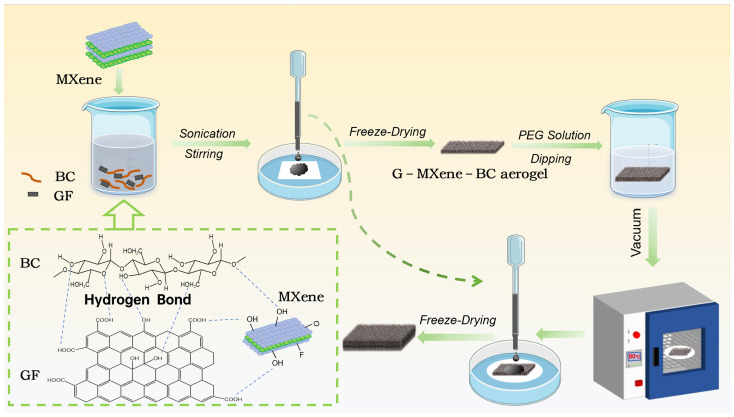
Preparation process diagram.

Figure 6 shows the preparation process of MXene-based self-heating thermoregulation. The delaminated MXene was synthesized by the wet chemical etching method. First, 0.8 g LiF and 0.5 g Ti_3_AlC_2_ powder were added into 10 mL of HCl solution and stirred for 24 h at 35 °C to extract Al. The etching and exfoliation conditions were optimized based on previous work [42]. The mixture was centrifuged at 10,000 rpm for 12 min; after that, the sediment was dispersed in DI water, followed by sonication for 30 min. The MXene suspension was then centrifuged at 3500 rpm for 40 min to obtain the uniform delaminated MXene colloidal solution. Finally, the resultant solution was collected and freeze-dried for 48 h.

#### 4.1.3. Preparation of the Monolayer G–MXene–BC Aerogel

The graphene oxide aqueous dispersion (10 mL), bacterial cellulose dispersion (6.25 g), and MXene dispersions with different masses (25 mg, 50 mg, and 100 mg) were added to deionized water (10 mL), and then the solution was sonicated, mixed, and stirred well. After that, the mixed solution was applied to the polymer basement membrane, and the G–MXene–BC aerogel was obtained by freeze-drying.

#### 4.1.4. Fabrication of G-M-BC/PEG Composite Aerogels by Combining Phase Change Material PEG

First, 35 g of transparent PEG-2000 liquid was obtained by magnetic stirring at 80 °C. Second, the G–MXene–BC aerogel was immersed in the PEG solution and then placed in a vacuum oven at 80 °C for 60 min. Following this, the aerogel was extracted and positioned on filter paper. Then, after an additional 4 h of vacuum drying, the monolayer G–MXene–BC/PEG composite phase change film was successfully synthesized and designated as 1s-G-M-BC/PEG (with a MXene content of 50 mg). Analogous to the above process, a bilayer MXene-based aerogel composite phase change film was fabricated by applying an additional layer of a mixture containing MXene, GO, and BC at the same concentration, followed by freeze-drying. Based on the MXene concentration, the formulations were designated as follows: 1d-G-M-BC/PEG (with 50 mg of MXene), 0.5d-G-M-BC/PEG (with 25 mg of MXene), and 2d-G-M-BC/PEG (with 100 mg of MXene).

#### 4.1.5. Characterization

The microscopic morphology and element distribution of G-M-BC/PEG composite phase change films were measured using X-ray diffraction patterns (XRD, Thermo Scientific, Gemini 300, Waltham, MA, USA) and scanning electron microscopes (SEM, ZEISS, S-4800, Oberkochen, Germany). The surface chemistry of the samples was obtained using an X-ray photoelectron spectrometer (XPS, Rigaku, K-Alpha, Tokyo, Japan). The mechanical properties of G-M-BC/PEG composite phase change films were tested using a universal mechanical testing machine (INTRON, 5982, Norwood, MA, USA). The thermal storage properties and cycling stability of the composite phase change films were tested by differential scanning calorimetry (DSC, LINSEIS, DSC PT 10, Selb, Germany). The photothermal conversion properties were monitored using infrared thermal imaging (FOTRIC, 225S, Shanghai, China) under one sun irradiation (100 mW cm^−2^) using a UV deuterium light source (NBET, NBET-D3000, Beijing, China) with an output spectral range of 160–400 nm.

### 4.2. Applications of the G-M-BC/PEG Composite Aerogels in the Warm-Up Transition Phase of the 200-Meter Sprint

#### 4.2.1. Test Indicators

Focusing the application on the widespread warm-up transition in sports exercise training, the G-M-BC/PEG composite phase change film can be used as a thermoregulation wearable device to achieve a personal heat management function. By testing exercise metrics and electromyography (EMG) indices, this study analyzes the multidimensional impacts of the thermoregulatory wearable device on subjects, including their subjective sensations, warm-up effects, 200 m sprint performance, and muscle movement status.

#### 4.2.2. Procedures

The experiments were carried out on an outdoor track and field under the same environmental conditions (10~15 °C air temperature). For adequate sunlight, all the procedures took place at 11:00 AM~1:00 PM on a sunny day. High-level athletes were employed as subjects for this study. Eight athletes (males; age 20.5 ± 1.93 yrs; stature 182.38 ± 3.81 cm; 75 ± 5.21 kg) were tested in a balanced crossover design under two interventions: wearing thermoregulation wearable film (Group A) and not wearing it (Group B), a condition serving as an internal control. Each subject repeated the experiment twice, 72 h apart, and was requested to refrain from consuming caffeine or alcohol and to avoid any strenuous exercise in the 24 h prior to testing (Figure 5a; Video S1).

## Data Availability

All data generated or analyzed in this study are included in the article; further inquiries can be directed to the corresponding authors.

## Supplementary Materials

The following supporting information can be downloaded at: https://www.mdpi.com/article/10.3390/gels12040320/s1, Video S1: 200-m sprint test with EMG signal acquisition procedure; Table S1: Physiological indicators collected during the 200-m sprint test; Table S2: The 200-m timed race and split times for Group A (wearing group) and Group B (non-wearing group) (*n* = 8); Table S3: Muscle fatigue index in Group A and Group B (*n* = 8); Table S4: RMS of lower limb muscles during muscle activity in Group A and Group B (*n* = 8); Table S5: RMS of lower limb muscles during muscle fatigue in Group A and Group B (*n* = 8); Table S6: Muscle fatigue index in Group A and Group B (*n* = 8); Table S7: Scalability assessment of the G-M-BC/PEG composite film.
