# Supplementary material for: Preparation and Evaluation of MXene/Graphene-Integrated Cellulose Aerogel Composite for Self-Heating Thermoregulation in Athletic Warm-Up Optimization
Source: Gels. 2026 Apr 8;12(4):320. doi: 10.3390/gels12040320 (PMC13116451; doi:10.3390/gels12040320)
Supplement: Supplementary file 1 [file gels-12-00320-s001.zip › gels-4201463-supplementary.pdf]

Electronic Supplementary Material

Preparation and Evaluation of MXene/Graphene-Integrated Cellulose Aerogel Composite for self-heating Thermoregulation in Athletic Warm-up Optimization

Video S1: 200-meter sprint test with EMG signal acquisition process. (The researchers collected EMG data by following the running subjects on an electric bike.)

Table S1: Physiological indicators collected during the 200-meter sprint test.

Table S1 T_{skin}, RPE, thermal sensation and thermal comfort values recorded pre-warm-up, during the warm-up transition phase and after the 200m sprint time trial

		Pre-warm-up	Warm-up transition phase				After 200m sprint
			Immediately	5 min	10 min	15 min	
T_{skin} (℃)	A	31.73±1.73	31.99±1.21	33.29±2.60*	33.66±1.84*△	33.95±1.75*△	30.80±1.50
	B	31.42±1.24	31.66±1.67	32.33±1.48	31.41±1.42	32.04±2.40	29.53±2.79△
RPE	A	12±2.78	12.375±2	11.5±2.51	11.5±2.62	11.375±2.62	16.875±1.96
	B	11.625±2.83	11.875±1.46	12±1.31	11.625±1.85	11.875±1.89	15.125±2.75
Thermal sensation	A	-0.50±0.76	1.25±0.89*	1.50±0.54*	1.75±1.04*	2.00±1.31*△	1.25±1.17*
	B	-1.50±0.93	1.00±1.07*	0.63±1.51*	-0.12±1.55*	0.13±1.89*	0.63±1.85*
Thermal comfort	A	0.38±0.74	0.25±0.46	0.13±0.35	0.13±0.35	0.13±0.35	0.50±0.76
	B	0.88±1.36	0.38±0.74	0.63±0.92	0.75±1.04	1.00±1.41	0.62±1.41
Heart rate	A	77.25±11.89	94.87±12.28*	87.13±6.29*	84.00±90.13*△	82.75±14.10*△	140.00±24.04*△
	B	69.63±11.60	86.12±19.15*	74.00±12.67*△	73.63±90.67*△	73.5±13.84*△	135.50±33.02*△

* indicates a significant difference to the baseline within the group; Δ indicates a significant difference to the immediate after-warm-up result within the group ($p<0.05$)

Muscle movement state data analysis The EMG signals were bandpass-filtered at 5-500 Hz with a fourth-order Butterworth bandpass filter, followed by full-wave rectification. A 500 ms time window was used as the calculation width, and the moving average amplitude of the EMG signals from the MVC test and the maximum value were used to normalize the EMG signals during the 200m sprint time trial.

After full-wave rectification, a 500 ms time window used as the calculation width, and the Root Mean Squared Value (RMS) was calculated. The valid values for the first 5 seconds of the 200m sprint were intercepted as the EMG active phase, and values for the 5 seconds after the 150m time were taken as the EMG fatigue phase. The average RMS amplitude was calculated for the tested muscles in each phase, and the RMS

maxima were normalized by the surface EMG signals recorded during the MVC test, and then further fatigue index calculations were performed.

RMS:

$$\mathrm{RMS} = \frac{\mathrm{sqrt}\left(\sum_{i=0}^{n} EMG_i^2\right)}{N} \quad \text{(S1)}$$

where EMGi2 is the square of the myoelectric amplitude of the muscle at the i-th sampling point and N is the data length of the calculated RMS.

RMS normalized formula:

$$\mathrm{RMS}_{Normalized} = \frac{RMS_{raw}}{RMS_{MVC}} \quad \text{(S2)}$$

where RMSNormalized and RMSraw refer to the RMS after and before the normalization in the 200m sprint and RMSmvc is a normalized value based on the EMG signals recorded during the MVC test.

Fatigue index：

$$\text{Fatigue index} = \frac{RMS_{LastEpoch}}{RMS_{FirstEpoch}} \quad \text{(S3)}$$

where RMSFirstEpoch is the RMSNormalized in the first 5 seconds of the 200 time trial and RMSLastEpoch is the RMSNormalized for 5 seconds after the subject has run 150m.

Table S2: Figure 5. (b) The 200m sprint time trial performance times in both conditions.

Data are presented as mean ± standard deviation.

As shown in Table S2, there was a significant difference in the 200-meter time trial results between Group A and Group B ($P = 0.039 < 0.05$). However, there were no significant differences between the groups in the 100-meter split times ($P = 0.117 > 0.05$) or the 150-meter split times ($P = 0.109 > 0.05$). The results indicate that the use of smart temperature-regulating wearable devices has a certain impact on the athletic performance of college students in physical education classes.

Table S2: The 200-meter timed race and split times for Group A (wearing group) and Group B (non-wearing group) (n=8)

	200m sprint time (seconds)	100m sprint time (seconds)	150m sprint time (seconds)
Group A	25.46±1.01[a]	12.67±0.28	19.03±0.74
Group B	26.51±0.59[a]	13.27±1.05	19.59±1.17

[a] indicates a statistically significant difference between groups ($P < 0.05$)

Table S3. Figure 5. (c) Change in blood lactate (La^{-}) from baseline (pre-warm-up) to 10 min after a 200 m sprint, for both conditions.

Data are presented as mean ± standard deviation.

As shown in Table S3, there was a significant difference between the 200m time trial results of Group A and Group B ($P=0.039 < 0.05$). The segment score of 100m was ($P=0.117>0.05$) and the segment score of 150m was ($P=0.109>0.05$). There was no significant difference between the groups.

Table S3: Muscle fatigue index in Group A and Group B (n=8)

	Group A	Group B
Blood lactate (mmol/L)		
Pre-warm up	4.00±2.66	3.83±1.91
Immediately after 200m	13.73±5.76	11.66±4.33
3 minutes after 200m	13.45±3.93	11.41±5.30
5 minutes after 200m	13.13±3.85	9.16±2.60
7 minutes after 200m	11.33±2.00	10.45±4.35
10 minutes after 200m	11.24±2.62	11.14±5.18

Table S4. Figure 5. (d) RMS during the EMG active phase.

Data are presented as mean ± standard deviation.

As shown in Table S3, the order of lower limb muscle activation between Group A and Group B is not entirely consistent. The order for Group A is vastus lateralis (2.82 ± 1.42) > rectus femoris (2.27 ± 1.64) > vastus medialis (1.72 ± 0.88) > semitendinosus (1.65 ± 0.41) > gastrocnemius (1.37 ± 0.83) > long head of the biceps femoris (1.35 ± 0.66). The order for Group B is rectus femoris (2.83 ± 1.60) > long head of the biceps femoris (2.30 ± 2.10) > gastrocnemius (2.25 ± 2.09) > vastus lateralis (2.11 ± 0.91) > vastus medialis (2.02 ± 1.12) > semitendinosus (1.97 ± 1.42). Although the order of activation is not entirely consistent, in the early muscle activation phase of the 200m race for both Group A and Group B, the rectus femoris and vastus lateralis on the anterior thigh are the muscles with higher activation levels. Data analysis shows that there is no significant difference in the RMS values of the various muscles during the muscle activation phase between Group A and Group B.

Table S4: RMS of lower limb muscles during muscle activity in Group A and Group B (n=8)

	Group A	Group B
RMS		
VM	1.72±0.88	2.02±1.12
VL	2.82±1.42	2.11±0.91
RF	2.27±1.6	2.83±1.6
BF	1.35±0.66	2.30±2.1
ST	1.65±0.41	1.97±1.42
IG	1.70±1.06	2.25±2.09

Table S5. Figure 5. (e) RMS during the EMG fatigue phase.

Data are presented as mean ± standard deviation.

Figure S5 shows that during the muscle fatigue phase, the order of lower limb muscle activation still did not fully align between Group A and Group B participants. The order for Group A was vastus lateralis (3.03 ± 1.50) > gastrocnemius (2.63 ± 2.24) > rectus femoris (2.22 ± 1.69) > adductor longus (1.87 ± 0.83) > semitendinosus (1.53 ± 0.70) > long head of the biceps femoris (1.49 ± 0.79). The order for Group B was rectus femoris (2.92 ± 1.57) > gastrocnemius (2.46 ± 2.36) > vastus lateralis (2.26 ± 0.98) > long head of the biceps femoris (2.08 ± 1.81) > vastus medialis (2.04 ± 0.99) > semitendinosus (1.82 ± 1.23). The results show that there were no significant differences in RMS values among the muscles in Groups A and B during the muscle fatigue phase. The order of muscle activation was generally similar to that during the muscle active phase and exhibited an increasing trend in numerical values.

Table S5: RMS of lower limb muscles during muscle fatigue in Group A and Group B (n=8)

	Group A	Group B
RMS		
VM	1.87±0.83	2.04±0.99
VL	3.03±1.51	2.26±0.98
RF	2.22±1.69	2.92±1.57
BF	1.49±0.79	2.08±1.81
ST	1.53±0.70	1.82±1.23
IG	2.63±2.24	2.46±2.36

Table S6. Figure 5. (f) Statistical histograms of FI for each muscle.

Data are presented as mean ± standard deviation.

The fatigue index was calculated by dividing the normalized RMS of the 200 m terminal muscle fatigue phase by the normalized RMS of the 200 m pre-active muscle phase.

Table S6: Muscle fatigue index in Group A and Group B (n=8)

	Group A	Group B
Fatigue Index		
VM	1.13±0.18	1.09±0.23
VL	1.08±0.23	1.08±0.19
RF	1.02±0.20	1.14±0.48
BF	1.08±0.24	0.93±0.12
ST	0.90±0.25	0.97±0.20
IG	1.35±0.52	1.09±0.19

Table S7. Scalability assessment of the G-M-BC/PEG composite film

Process step	Materials/operation involved	Scalability potential	Main challenges for scale-up	Possible optimization strategies
MXene synthesis	Ti_3AlC_2, LiF, HCl	Moderate	Chemical handling, etching safety, batch consistency, waste treatment	Optimize etching conditions, improve yield, adopt safer and standardized MXene preparation protocols
GO/MXene/ BC mixing	GO dispersion, MXene dispersion, BC dispersion	High	Uniform dispersion and interfacial consistency in large batches	High-shear mixing, dispersion monitoring, standardized loading
Aerogel framework formation	GO–MXene–BC porous matrix	Moderate	Structural uniformity and pore consistency at larger dimensions	Process control for gelation, freezing and drying; modular fabrication routes
PEG impregnation	Porous scaffold encapsulated with PEG-2000	High	Leakage control, cycle-to-cycle reproducibility	Vacuum-assisted impregnation, optimized PEG loading, interface engineering
Film application	Wearable assembly	High	Mechanical durability, skin conformity, long-term use stability	Flexible packaging, repeated-use validation
